# Hypoxia favors myosin heavy chain beta gene expression in an Hif-1alpha-dependent manner

**DOI:** 10.18632/oncotarget.19016

**Published:** 2017-07-05

**Authors:** Lucia Binó, Jiřina Procházková, Katarzyna Anna Radaszkiewicz, Jan Kučera, Jana Kudová, Jiří Pacherník, Lukáš Kubala

**Affiliations:** ^1^ Institute of Biophysics of the CAS, Brno, Czech Republic; ^2^ Institute of Experimental Biology, Department of Physiology and Immunology of Animals, Faculty of Science, Masaryk University, Brno, Czech Republic; ^3^ Department of Histology and Embryology, Faculty of Medicine, Masaryk University, Brno, Czech Republic; ^4^ International Clinical Research Center, Center of Biomolecular and Cellular Engineering, St. Anne's University Hospital Brno, Brno, Czech Republic

**Keywords:** mouse, heart, myosin heavy chain, fetal gene program, hypoxia

## Abstract

The potentiation of the naturally limited regenerative capacity of the heart is dependent on an understanding of the mechanisms that are activated in response to pathological conditions such as hypoxia. Under these conditions, the expression of genes suggested to support cardiomyocyte survival and heart adaptation is triggered. Particularly important are changes in the expression of myosin heavy chain (MHC) isoforms. We propose here that alterations in the expression profiles of MHC genes are induced in response to hypoxia and are primarily mediated by hypoxia inducible factor (HIF). In *in vitro* models of mouse embryonic stem cell-derived cardiomyocytes, we showed that hypoxia (1% O_2_) or the pharmacological stabilization of HIFs significantly increased MHCbeta (*Myh7*) gene expression. The key role of HIF-1alpha is supported by the absence of these effects in HIF-1alpha-deficient cells, even in the presence of HIF-2alpha. Interestingly, ChIP analysis did not confirm the direct interaction of HIF-1alpha with putative HIF response elements predicted in the MHCalpha and beta encoding DNA region. Further analyses showed the significant effect of the mTOR signaling inhibitor rapamycin in inducing *Myh7* expression and a hypoxia-triggered reduction in the levels of antisense RNA transcripts associated with the *Myh7* gene locus. Overall, the recognized and important role of HIF in the regulation of heart regenerative processes could be highly significant for the development of novel therapeutic interventions in heart failure.

## INTRODUCTION

The sensitivity of the mammalian myocardium to pathological alterations is related to its highly limited regenerative potential [1, 2]. Interestingly, in a variety of pathophysiological conditions including ischemia, hypertrophy, and atrophy, a common response of the heart is the induction of fetal gene reprogramming associated with complex changes in gene expression profiles, often suggested to support cell adaptation and survival [3]. These include modulation of the expression of genes coding for myosin heavy chains (MHCs). The expression of the α isoform of MHC (MHCα; encoded by the *Myh6* gene) decreases, while the expression of the β isoform (MHCβ; encoded by *Myh7* gene) increases. Physiologically, the ratio of MHC isoform expressions changes in the opposite manner after birth under the control of thyroid hormone [4–6]. The critical pathological event responsible for such regulation is not yet well understood; however, it has been suggested that it is most likely related to alterations in the myocardium microenvironment [7].

The acute switch from an aerobic to an anaerobic metabolism occurring in cardiac cells appears to be a necessary prerequisite for immediate cell survival during hypoxia, the oxygen deprivation of tissues and cells [7, 8]. The modulation of the energetic metabolism is suggested by various authors as an important factor in the induction of the evolutionarily conserved fetal gene program [7, 8]. Interestingly, a positive correlation between the glycolytic metabolism and MHCβ isoform expression has also been described in primary rat cardiomyocytes cultured *in vitro* [9].

The key regulatory factor involved in cell response to a decreased oxygen level is hypoxia-inducible factor (HIF) [10]. Transcriptionally active HIF is composed of two subunits; one of the three oxygen-dependent HIF-α subunits (HIF-1α, -2α, or -3α) forms dimers with HIF-1β [11, 12]. The most intensively studied subunit, HIF-1α, is upregulated by oxygen at two levels – protein stability and transcriptional activity – via prolyl hydroxylase domain enzymes (PHD1–3) and factor inhibiting HIF, respectively [13, 14]. Murine HIF-1α is expressed at high levels in the ventricular wall, but decreases throughout embryonal development [15–17]. There is no detectable level of HIF-1α protein in healthy adult ventricles [18]. Interestingly, a number of cardiac pathophysiologies are associated with increased HIF-1α [18]. An increase in HIF-1α mRNA levels is one of the earliest responses to myocardial ischemia [19]. Interestingly, hypoxia was suggested to decrease MHCα and to increase MHCβ transcript levels in rat *in vivo* and *in vitro* models [20]. Moreover, the pharmacological stabilization of HIF-1α, mediated by PHD inhibitor dimethyloxalylglycine (DMOG), was shown to be cardioprotective [21, 22].

Besides HIF-directed hypoxic responses, there are also several other hypoxia-related pathways which ensure ATP conservation by limiting energy-consuming processes in favor of processes that are critical for the maintenance of cell viability [23]. The mammalian (also mechanistic) target of rapamycin (mTOR) is a serine/threonine protein kinase which regulates protein translation, cellular metabolism, and the organization of actinin in response to nutrient, growth factors, and O_2_ availability [23, 24]. mTOR is inhibited in the majority of cells in response to hypoxia [23].

Notably, the expression of MHCs is currently understood to be repressed by antisense RNA molecules transcribed in the healthy myocardium [25–27]. Antisense *Myh7* RNA was shown to inhibit the processing of sense pre-mRNA into mature mRNA, thus turning off *Myh7* gene expression [26, 28]. In addition, the long noncoding RNA (lncRNA) Mhrt transcribed in the *Myh7* locus was recently reported to protect the heart from pathological hypertrophy [29].

Considering the association between changes in oxygen supply and the modulation of *Myh6* and *Myh7* gene expression, we suggest here that the primary trigger crucial for activation of the fetal gene program is the limited amount of oxygen. Uncovering the possible role of hypoxia, particularly reflected by the role of HIF-1α, in the regulation of MHC gene expression is therefore the main goal of this work.

## RESULTS

### Hypoxic conditions and the pharmacological stabilization of HIF induce MHCβ expression in cardiomyocytes derived from mESC

**Figure 1:**
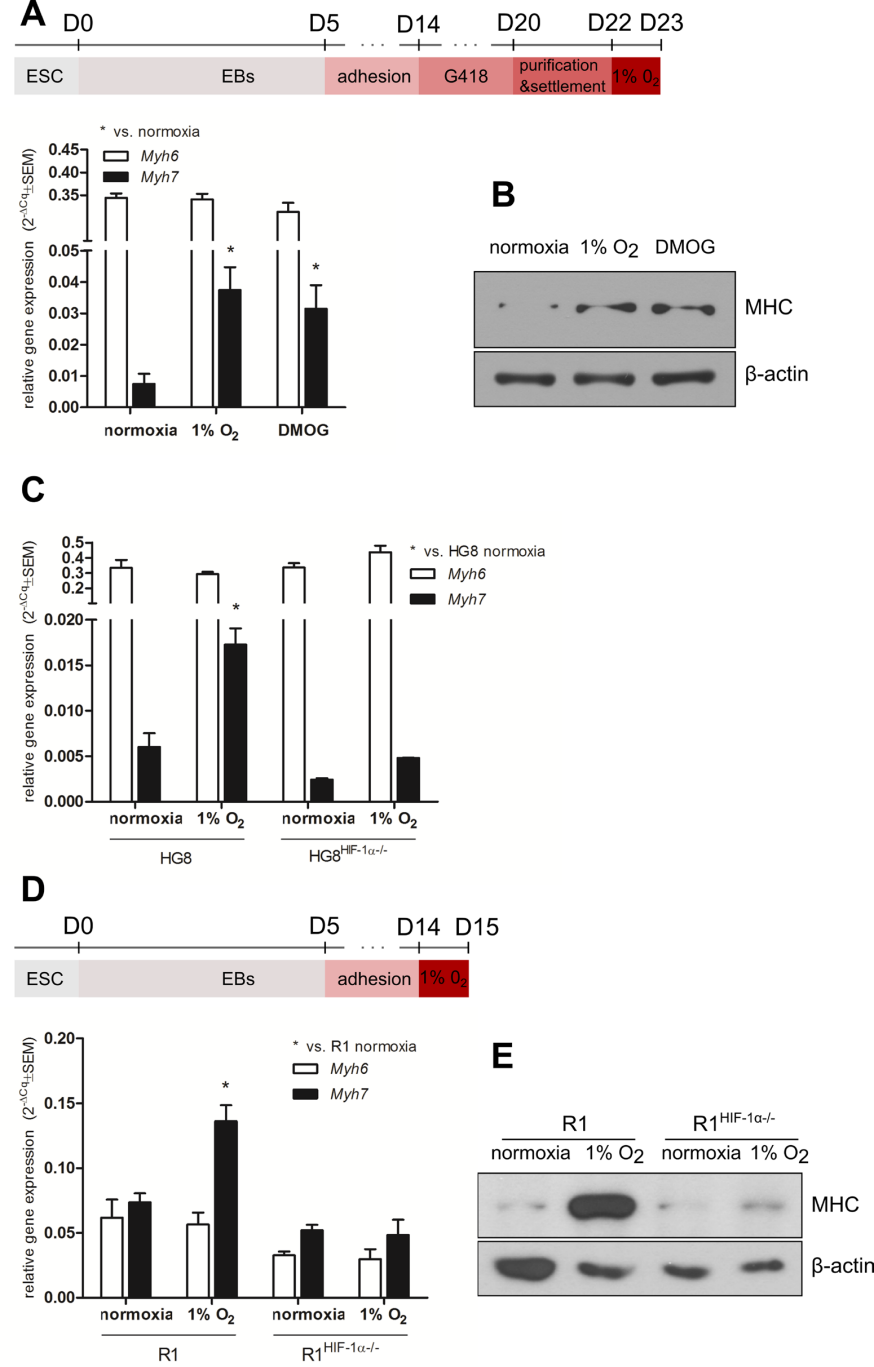
Effect of hypoxia and the pharmacological stabilization of HIF on MHC expression Time-scheme of cardiomyocyte derivation from A top) HG8 and D top) R1 and R1^HIF-1α−/−^ mESC. *Myh6* and *Myh7* relative mRNA levels in (**A**) cardiomyocytes derived from HG8 mESC (*n* = 3), (**C**) cardiomyocytes derived from parental HG8 and HG8^HIF-1α−/−^ mESC (*n* = 3), and (**D**) cardiomyocytes derived from R1 and R1^HIF-1α−/−^ mESC determined by qRT-PCR (*n* = 4 (R1), *n* = 3 (*R1^HIF-1α^*^−*/*−^)). Data are presented as 2^−ΔCq^ ± SEM (groups were compared using one-way ANOVA with the Tukey (HSD) post hoc test *p < 0.05). Total MHCα and β protein levels (**B**) in cardiomyocytes derived from HG8 mESC (*n* = 2) and (**E**) in cardiomyocytes derived from R1 and R1^HIF-1α−/−^ mESC (cropped representative western blots from total *n* = 2 are shown).

We engaged a model system represented by preselected (pMHCα-Neo-Hyg vector) cardiomyocytes differentiated *in vitro* from mESC (HG8) to observe the impact of hypoxic exposure. Hypoxia (1% O_2_) or the presence of DMOG, inducing the pharmacological stabilization of HIF-α subunits by the inhibition of PHDs [30], upregulated the gene expression of *Myh7* (Figure 1A, 1C) as well as total MHCα and β protein levels (Figure 1B) in comparison with normoxic cultivation. Aware of the fact that in the HG8 model the MHCα expression profile might be biased by cardiomyocyte pre-selection based on *Myh6* promoter activity, we also tested the expression of both cardiac MHC isoforms upon hypoxic exposure in the parental R1 cell line and again observed the upregulation of *Myh7* mRNA expression (Figure 1D) and total MHCα and β protein levels (Figure 1E).

### The hypoxia-induced upregulation of MHCβ expression is dependent on the presence of HIF-1α

**Figure 2:**
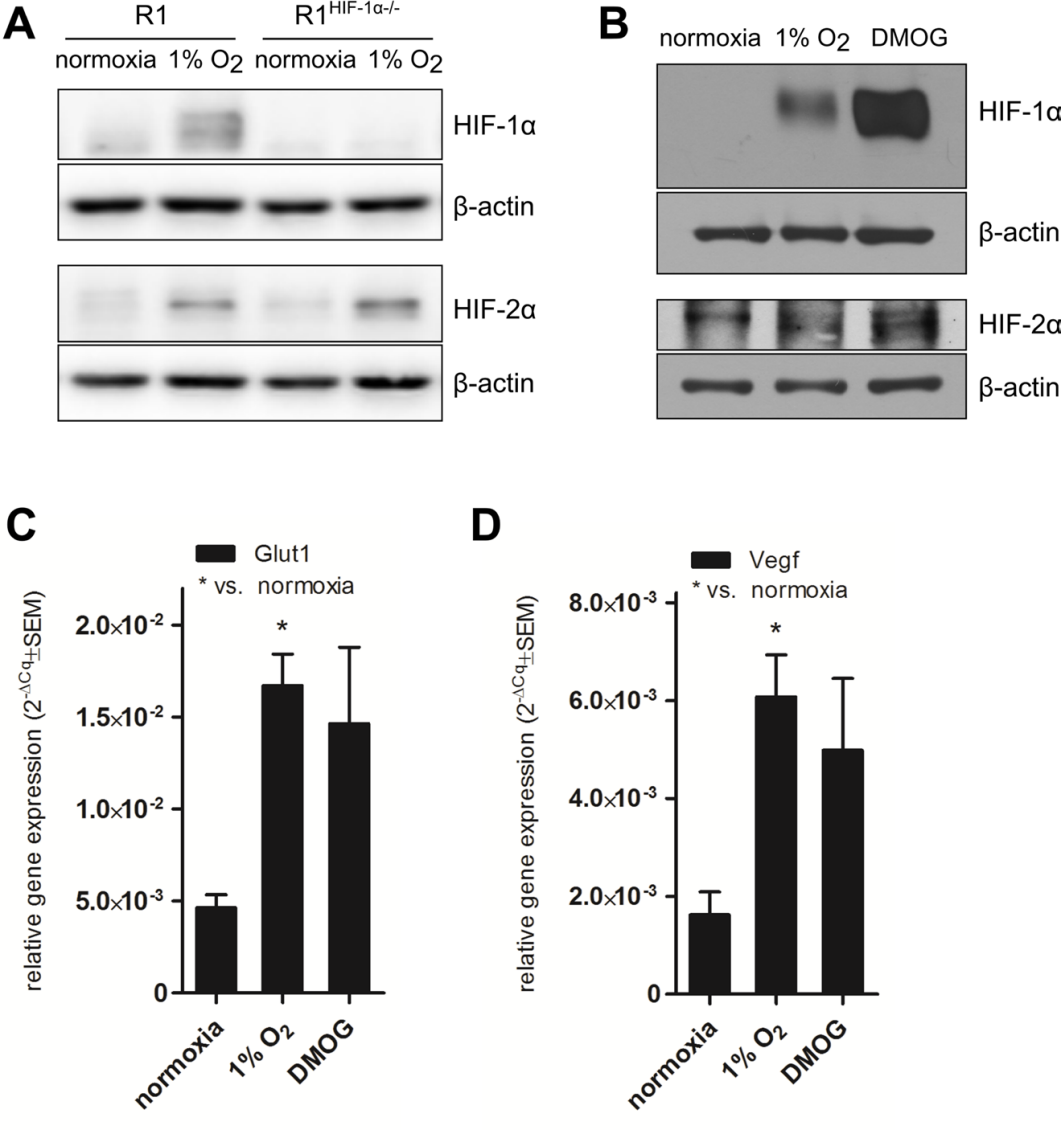
HIF protein stabilization and HIF-1α transcriptional activity Representative western blot showing protein levels of HIF-1α and HIF-2α (*n* = 3) in (**A**) cardiomyocytes derived from R1 and R1^HIF-1α−/−^ mESC exposed to 1% O_2_ and (**B**) cardiomyocytes derived from HG8 mESC exposed to 1% O_2_ and DMOG. Cropped representative western blots are shown from total of *n* = 3. mRNA levels of (**C**) Glut1 and (**D**) VEGF in cardiomyocytes derived from HG8 mESC exposed to 1% O_2_ or DMOG determined by qRT-PCR (*n* = 3). Data are presented as 2^−ΔCq^ ± SEM (groups were compared using one-way ANOVA with the Tukey (HSD) post hoc test *p < 0.05).

To confirm the significance of HIF-1α in the described phenomenon we employed HG8^HIF-1α−/−^ and R1^HIF-1α−/−^ cells deficient in HIF-1α. Interestingly, neither the induction of *Myh7* mRNA (Figure 1C, 1D, Supplementary Figure 1) nor the increase in total MHCα and β protein levels (Figure 1E) was observed in cardiomyocytes differentiated from these HIF-1α-deficient cells. The missing inducibility of *Myh7*/ total MHCα and β protein in the cell cultures derived from HG8^HIF-1α−/−^ and R1^HIF-1α−/−^ argues for the dependence of the observed effect on the presence of HIF-1α. Indeed, HIF-1α protein was stabilized in R1-derived cardiomyocytes exposed to hypoxia, but was missing in R1^HIF-1α−/−^-derived cells, as expected (Figure 2A). Accordingly, in HG8-derived cardiomyocytes, hypoxia- or DMOG-induced *Myh7/*total MHCα and β protein upregulation goes hand-in-hand with both HIF-1α and HIF-2α protein stabilization under hypoxic conditions, with even higher levels of both proteins observed in the case of DMOG (Figure 2B). The transcriptional activity of stabilized HIFs is documented by the increased expression of genes known to be under the control of HIF-1α, particularly Glut1 and VEGF (Figure 2C, 2D).

### Analysis of HIF-1α binding to the DNA locus encoding MHCα and MHCβ

**Figure 3:**
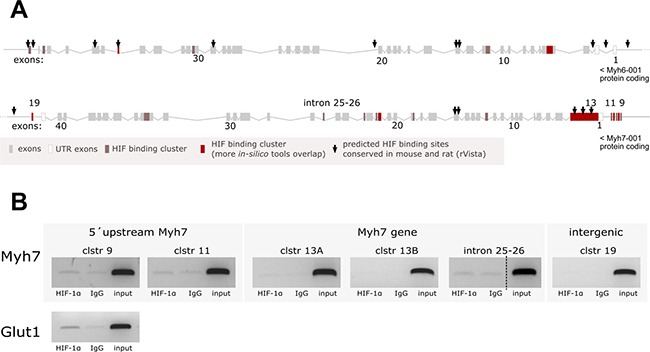
Analysis of HIF-1α binding to the DNA locus encoding *MHCα* and *MHCβ* (**A**) Graphical representation of predicted HIF binding sites in the locus encoding for mouse MHCα and MHCβ; sites conserved in mouse and rat are marked with arrows. (**B**) ChIP analysis of HIF-1α binding to predicted binding sites performed on samples from 10 day-differentiated R1 mESCs exposed to 1% O_2_ hypoxia for 24 h. HIF-1α binding to its target gene Glut1 served as ChIP assay positive control (cropped representative gels are shown from *n* = 4 (intron 25–26, Glut1) or *n* = 3 (other clusters)).

The observed correlation between HIF-1α protein stabilization and *Myh7/*total MHCα and β protein expression induced by hypoxia and DMOG (Figures 1, 2) encouraged us to test the hypothesis that HIF-1α might control either *Myh7* or *Myh6* expression via its direct binding to the putative HREs predicted in the corresponding DNA locus. We therefore performed *in silico* analysis of the ~100 kb-long DNA region encompassing mouse *Myh7* and *Myh6* genes and their adjacent UTR sequences. One putative HIF binding site in intron 25–26 of the *Myh7* gene and another 28 potential clusters of HIF binding sites were predicted by different web-based algorithms (Figure 3A; only clusters within the 10kb surroundings of the coding sequences are depicted; see details in section Materials and Methods). For the ChIP analysis, we selected clusters 9 and 11 (*Myh7* upstream), clusters 13a and 13b (*Myh7* encoding region), cluster 19 (intergenic region) and the putative HIF-binding site located in *Myh7* intron 25–26, i.e. HRE-rich regions identified by the majority of predictive algorithms. ChIP analysis of samples from 10-day-differentiated R1 mESCs exposed to 1% O_2_ hypoxia for 24 h, in which both HIF stabilization and MHC expression were observed (Figure 3B and Supplementary Figure 2), did not confirm the hypothesized direct binding of HIF-1α.

### Hypoxia and the pharmacological stabilization of HIF slow down the induction of *Myh6* in a mouse embryonal heart model

**Figure 4:**
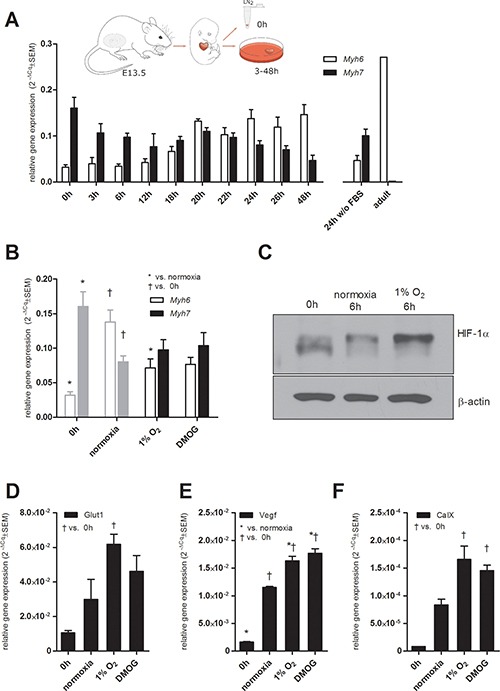
Mouse fetal heart model (**A**) Scheme showing the derivation of fetal hearts and kinetics of changes in *Myh6* and *Myh7* mRNA levels in mouse explanted fetal hearts cultured *in vitro* determined by qRT-PCR; for comparison, *Myh6* and *Myh7* mRNA levels in an adult heart sample are shown. Data are expressed as mean 2^−ΔCq^ ± SEM *Myh6* and *Myh7* (*n* = 10 (48 h), *n* = 7 (0 h, 24 h), *n* = 4 (3 h, 22 h, 2 h wo FBS), *n* = 3 (6 h, 12 h, 18 h, 20 h, 26 h)), *n* = 1 (adult heart). Effect of hypoxia and the pharmacological stabilization of HIF on (**B**) *Myh6* and *Myh7* relative mRNA levels determined by qRT-PCR (*n* = 9 (0 h), *n* = 7 (normoxia), *n* = 8 (1% O_2_), *n* = 5 (DMOG); same data depicted in Figure 4A are displayed in grey for comparison); (**C**) stabilization of HIF-1α protein after 6 h of *in vitro* culture in normoxia and 1% O_2_ (cropped representative western blots are shown, *n* = 3), mRNA levels of (**D**) Glut1, (**E**) Vegf, and (**F**) CaIX after 24 h determined by qRT-PCR (*n* = 3), data are presented as 2^−ΔCq^ ± SEM (groups were compared using one-way ANOVA with the Tukey (HSD) post hoc test *p < 0.05; ^†^*p* < 0.05).

To monitor the effect of hypoxia on the changes in the tested MHC expressions in heart tissue, we established a model derived from explanted mouse embryonal hearts (Figure 4A). Fetal mouse hearts predominantly express the MHCβ isoform, which is gradually displaced by the adult MHCα isoform after birth. Considering this and the previously described phenomenon that the expression of both cardiac MHC isoforms is regulated by thyroid hormone [4–6], we cultured excised hearts from 13.5-dpc-old mouse embryos *in vitro* in the presence of FBS containing thyroid hormone.

The kinetics of changes in *Myh6* and *Myh7* expression profiles was analyzed over time and clearly confirmed the induction of *Myh6* by FBS and the overall suppression of *Myh7* expression (Figure 4A). For comparison, levels of *Myh6* and *Myh7* expression profiles in hearts incubated in the absence of FBS and in an adult heart sample are shown (Figure 4A).

In order to further analyze hypoxia/HIF-mediated changes in the expression patterns of the tested MHCs, we exposed whole mouse fetal hearts to hypoxic conditions (24 h). Indeed, hypoxic treatment significantly delayed the increase in *Myh6* mRNA observed in fetal hearts cultivated in medium with FBS under normoxic conditions (Figure 4B). Furthermore, the pharmacological stabilization of HIF-α subunits by DMOG resulted in the same effect (Figure 4B).

In an effort to elucidate the role of HIF-1α and HIF-2α in this observation, we analyzed the protein levels of both transcription factors and the expression patterns of their target genes, *Glut1*, *CaIX* and *Vegf*. The stabilization of HIF-1α protein in hypoxia-exposed fetal hearts after 6 h (Figure 4C) was followed by the subsequent upregulation of mRNA levels of all three tested HIF targets after 24 h (Figure 4D–4F). Interestingly, analysis of the stabilization of HIF-1α protein after 24 h revealed lower levels of HIF-1α protein in samples incubated in hypoxia or with DMOG compared to control samples and also to samples from hearts without any incubation (Supplementary Figure 3). These data suggest that the observed regulatory effects of HIF-1α on MHC gene expression are driven by its transcriptional activity during the earlier phases of its stabilization by hypoxia or DMOG. Further, we were unable to credibly detect HIF-2α protein in fetal heart samples (data not shown).

This model of explanted hearts was also tested for HIF-1α binding to the DNA locus encoding MHCα and MHCβ. In agreement with the R1-derived model, no binding of HIF-1α in any of the six selected HREs-predicted clusters was detected by ChIP in fetal hearts incubated for 24 h in 1% O_2_ (*n* = 3, data not shown).

### mTOR inhibition increases *Myh7* expression in HG8-derived cardiomyocytes

**Figure 5:**
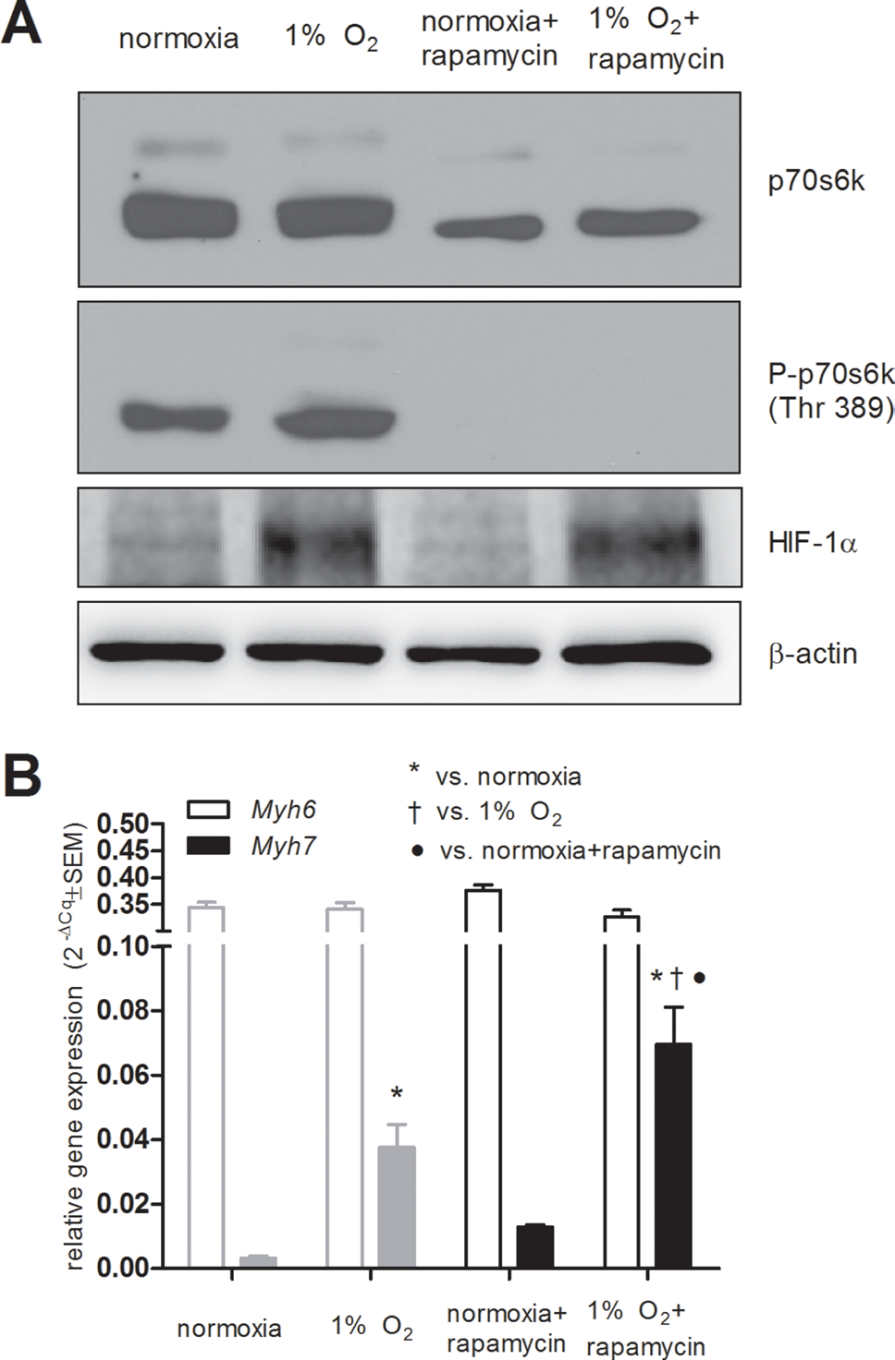
Effects of mTOR inhibition using rapamycin (10 ng/ml) (**A**) mTOR inhibition using rapamycin validated by western blot detection of its downstream target p70 s6 kinase (*n* = 6) and the stabilization of HIF-1α protein by hypoxia (*n* = 4) (**B**) *Myh6* and *Myh7* relative mRNA levels in normoxia and in 1 % O_2_ hypoxia determined by qRT-PCR. Data are presented as 2^−ΔCq^ ± SEM (*n* = 5 (normoxia), *n* = 4 (1% O_2_), *n* = 3 (rapamycin treatment); in grey same data depicted in Figure 1A are displayed for comparison; groups were compared using one-way ANOVA with the Fisher's LSD post hoc test *p < 0.05; ^†^*p* < 0.05).

Another possible mechanism previously shown to be engaged during hypoxia is mTOR signaling, which is known to be inhibited under hypoxic conditions. To test its role in our model, we employed treatment with rapamycin, an inhibitor of mTOR, in order to simulate hypoxia-related mTOR inhibition, and then we analyzed the expression of both *Myh*s in HG8-derived cardiomyocytes. Interestingly, we did not observe the downregulation of phosphorylation of its downstream target p70s6 kinase (Thr 389) in hypoxic conditions (Figure 5A). Rapamycin-induced mTOR inhibition was validated by the absence of phosphorylated p70s6k (Figure 5A). This resulted in the upregulation of *Myh7* expression, even in cardiomyocytes cultivated under normoxic conditions; however, this effect does not reach statistical significance (Figure 5B). Interestingly, the combination of rapamycin and hypoxic conditions induced the expression of *Myh7* even more potently than hypoxia itself (Figure 5B). As expected, HIF levels were stabilized after incubation under hypoxic conditions (Figure 5A).

### Hypoxia induces a decrease in antisense *Myh7* RNA transcript and Mhrt lncRNA level

**Figure 6:**
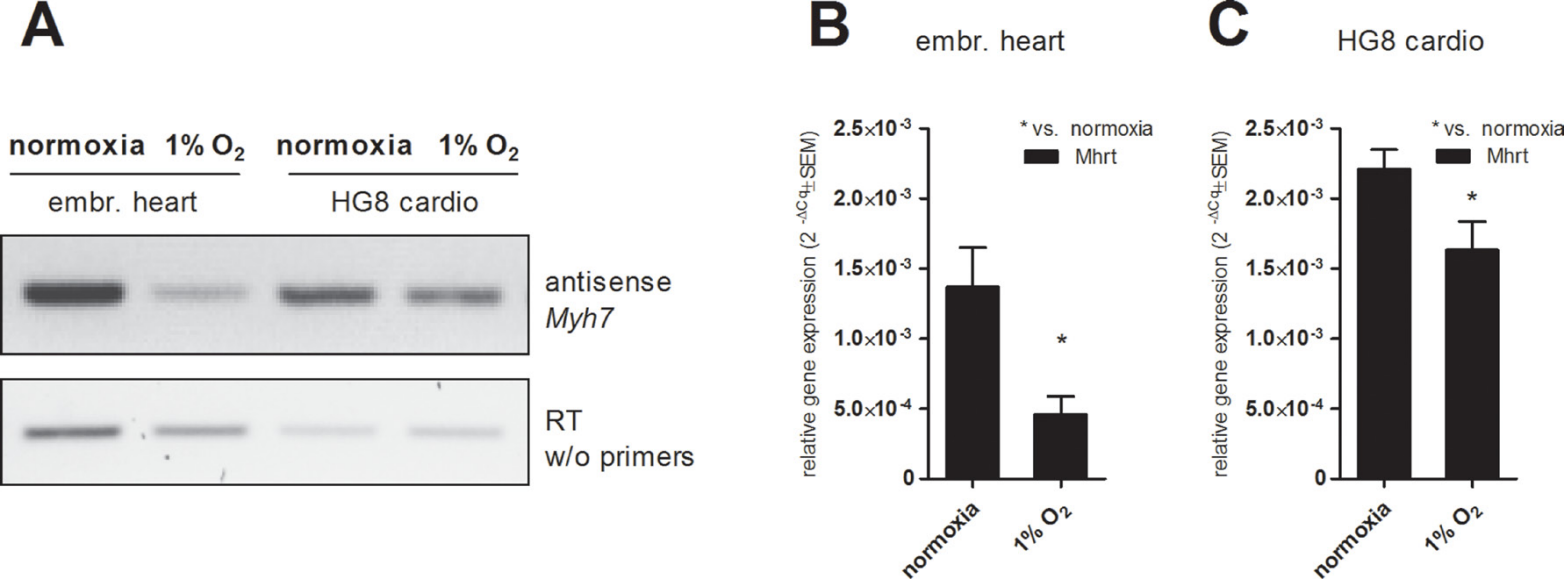
Effect of hypoxic exposure on the expression levels of (**A**) antisense *Myh7* transcript in fetal hearts and HG8-derived cardiomyocytes analyzed by antisense PCR and subsequent agarose electrophoresis (a template from the reverse-transcription without primers was used as a negative control (cropped representative gels are shown, (*n* = 3)) and of Mhrt transcript in (**B**) fetal hearts and (**C**) HG8 derived-cardiomyocytes determined by qRT-PCR. Data are presented as 2^−ΔCq^ ± SEM (*n* = 7 (embr. hearts 1% O_2_, HG8 normoxia), *n* = 4 (HG8 1% O_2_), *n* = 5 (embr. hearts normoxia); groups were compared using one-way ANOVA with the Tukey (HSD) post hoc test *p < 0.05).

In the light of recent discoveries concerning the regulation of MHC gene expression by naturally present antisense transcripts and lncRNAs, we tested the effect of hypoxia on the level of antisense *Myh7* transcript and Mhrt lncRNA. In both tested models (explanted fetal hearts and HG8-derived cardiomyocytes), hypoxia downregulated the levels of antisense *Myh7* transcript (Figure 6A) as well as RNA levels of *Mhrt* lncRNA (Figure 6B, 6C).

## DISCUSSION

Herein, we reveal the hypoxic regulation of MHC expression in cardiomyocytes, specifically the expression of the MHCβ isoform. We show for the first time that the effect of hypoxia on the cardiac MHC expression profile is HIF-1α-dependent, as no significant changes in MHC expression profiles were observed in HIF-1α-deficient cells exposed to hypoxia, even in the presence of HIF-2α. In all three presented models, HIF-1α protein was stabilized under hypoxic conditions and also transcriptionally active, according to HIF-target gene expression data measured in both the *in vitro* and the *ex vivo* model.

In line with this observation, hypoxia was indeed previously shown to increase MHCβ transcript levels in rat models *in vivo* (in rats exposed to 24 and 48 h of hypobaric hypoxia (11 % oxygen)) and *in vitro* (in neonatal rat cardiomyocytes incubated for up to 48 h in a hypoxic chamber) [20]. Further, justifying our hypothesis, hypoxia was previously shown to stimulate the expression of muscle slow-oxidative type I myosin in an HIF-1α dependent manner [31]. Although *Razeghi et al.* also showed a hypoxia-induced decrease in *Myh6* transcript levels in the rat models specified above, we did not observe any significant effect of hypoxia or DMOG on the *Myh6* expression profile in our mESC cell-derived models of cardiomyocytes [20]. This putative discrepancy could be explained by biased *Myh6* expression levels in HG8-derived cardiomyocytes purified on the basis of *Myh6* promoter activity and by the obvious partial variation of mESC-derived *in vitro* models and the *in vivo* situation. We therefore decided to study the negative effect of hypoxia (and DMOG) on *Myh6* transcript expression rather in the context of real cardiac tissue, and we, indeed, observed the delayed onset of FBS-induced *Myh6* upregulation in explanted mouse fetal hearts exposed to hypoxia and also DMOG. Notably, the *Myh7* expression pattern in hypoxia- and DMOG-treated explanted hearts tended to be slightly enhanced; however, this effect was only mild and did not pass the criteria of significance of the employed statistical method. Interestingly, lower levels of HIF-1α were detected in samples incubated in hypoxia or with DMOG compared to control samples after 24 h suggesting the observed regulatory effects of HIF-1α on MHC gene expression to be driven by HIF-1α transcriptional activity during the earlier phases of HIF-1α stabilization. We can speculate that increased levels of HIF-1α in samples incubated in normoxia for 24 h can reflect stress response in explanted hearts.

Since hypoxia is a condition common in fetal heart development and the failing adult heart, HIF-1α is a good candidate for being the regulator responsible for the postnatal switch and the return to the fetal gene program. Considering this, we decided to address the possibility of the *Myh6* and *Myh7* genes being direct gene targets of HIF-1α. However, we did not observe the direct binding of HIF-1α protein to either of the tested binding sites predicted in the corresponding DNA locus. Several considerations, however, must be kept in mind. First, the existence of a distant regulatory motif sensitive to the presence of HIF-1α cannot be excluded. Second, no reliable genome-wide study dedicated to the mapping of HIF-1α binding sites in cardiomyocytes exposed to hypoxic stress has been published so far. Nevertheless, the proposed indirect effect of HIF-1α on the induction of *Myh7* mRNA is strongly supported by the observation of no significant changes in *Myh7* gene expression during the short-term exposure of HG8-derived cardiomyocytes to hypoxia or DMOG (6 h) (data not shown). Interestingly, although we observed statistically significant hypoxia- or DMOG-mediated slowdown of FBS-induced *Myh6* upregulation in the model of mouse explanted hearts, a published suggestion based on another model that HIF-dependent gene suppression is almost entirely indirect [32] discouraged us from testing direct HIF-1α binding in genomic DNA regions flanking and corresponding to the *Myh6* gene. Thus, the involvement of other mechanisms triggered by hypoxia and/or HIF-1α protein stabilization has to be considered, these acting as potential regulators of MHC gene expression.

mTOR complex 1 (mTORC1) was previously shown to be necessary for embryonic cardiovascular development and for the postnatal maintenance of cardiac structure and function as well as for adaptation to pressure overload and the development of compensatory hypertrophy [33]. It is suggested that mTOR inhibition decreases the translation of the HIF-α subunit in mild hypoxia or even normoxia [34, 35] and HIF-1α is suggested to be responsible for glycolytic mTORC1 action [36]. However, hypoxic mTORC1 regulation is suggested to be HIF-1α-independent [37], and, notably, the HIF-2α-dependent regulation of mTORC1 activity has been proposed [38]. Interestingly, rapamycin was shown to have significant benefits for cardiovascular function [39] similarly to DMOG [21, 22]. Herein, we revealed that the inhibition of mTOR by rapamycin induces an increase in *Myh7* mRNA level similarly to the presence of hypoxia or DMOG. However, under our conditions, mTOR signaling was probably not responsible for the increase in *Myh7* mRNA level observed after the exposure of HG8-derived cardiomyocytes to hypoxia, since no change in the phosphorylated/active status of p70 s6 kinase, its downstream target, was simultaneously detected. The lack of inhibition of p70s6k phosphorylation by exposure to hypoxia in our model can be related to different kinetics and to a lower level of intensity of the inhibition of p70s6k phosphorylation in response to the employed level of hypoxia than in response to rapamycin. We can speculate that prolonged and more severe hypoxia would have a more profound inhibitory effect on p70s6k phosphorylation. The limited nature of the effect of the employed hypoxic conditions in our model is further supported by the observation that the combination of rapamycin and hypoxic conditions induced the expression of Myh7 more potently than hypoxia itself. Interestingly, Knaup et al. found that under severe hypoxia, no influence of mTOR inhibitors was observed; thus, the stimulation of HIF1α by mTOR may only be relevant under mild hypoxia or even normoxia [34].

Further, HIF-1α was previously shown to maintain the expression of the sarcomeric protein titin, but not that of the sarcomeric proteins EH-myomesin and α-actinin, during cardiac development [17]. Under conditions promoting changes in the tested MHC expression profiles we did not observe changes in sarcomeric α-actinin mRNA levels when compared to other reference gene Rpl13a (data not shown).

Significantly, and for the first time, we show here the hypoxia induces a decrease in *Myh7* antisense transcript. Antisense *Myh7* RNA was also found to be significantly decreased in 12-day pressure-overloaded hearts [26, 40]. Moreover, the relative level of MHC-associated RNA transcript (Mhrt), previously identified as the lncRNA molecule transcribed from *Myh7* loci and proposed as a cardioprotective molecule [29], was shown here to decrease in response to hypoxia. Interestingly, alterations in miRNA expression in heart failure were also reported to display a pattern similar to that observed in the fetal heart [41]. Thus, the role of other regulatory RNAs in MHC gene expression control remains to be elucidated.

Importantly, we also have to consider the difference between the regulation of MHC gene expression in humans and rodents. In humans, with slower heart rates than mice or rats, MHCβ is the predominant isoform throughout their whole life [42]. However, the ratio of MHCα to MHCβ isoforms is reduced in failing human ventricles, similarly as in the mouse heart during pathological processes [3, 43–46].

In conclusion, since ChIP analysis did not confirm direct interaction between HIF-1α and the proposed HREs located in the MHCα and MHCβ encoding DNA regions, we suggest that other hypoxia-associated and HIF-1α-dependent mechanisms are involved in the regulation of *Myh7* gene expression; in particular, the role of antisense RNA molecules remains to be elucidated. Another interesting finding is that the presence of HIF-2α itself is not sufficient to rescue the hypoxia-mediated induction of *Myh7*, as shown in the R1 and R1^HIF-1α−/−^ models. However, as discussed above, several other mechanisms which seem to regulate MHC isoform expression in heart have previously been described [29, 47]. The question is whether all these mechanisms compete or play a collective role, whether they are independent or synchronized by one upstream master regulator, and whether the overall mechanism works through the induction of repressors or by a decrease in stimulation. This knowledge could further help to elucidate the mechanism, when the adult heart returns to the fetal gene program under pathophysiological conditions, and possibly help to identify novel therapeutic targets for the treatment of heart failure.

## MATERIALS AND METHODS

### Mouse embryonic stem cell (mESC) culture

Feeder-free adapted maternal line R1, R1^HIF-1α−/−^ (derived from R1, the kind gift of Dr. P. Carmeliet [48]) and HG8 (derived from the R1 line transfected with pMHC-Neo-Hyg vector, the kind gift of Dr. Loren J. Field) mESC cell lines were propagated in an undifferentiated state as described earlier [49]. Briefly, mESC were cultured on gelatinized tissue culture plastic in Dulbecco's modified Eagle's medium (DMEM) containing 15% fetal calf serum (ESC tested; both from Invitrogen, USA), 1 × non-essential amino acid, 0.05 mM β-mercaptoethanol, 100 IU/ml of penicillin, and 0.1 mg/ml of streptomycin (all from PAA Laboratories, Austria), supplemented with 1 000 U/ml of leukemia inhibitory factor (LIF; Chemicon, USA), here referred to as the ES medium [30, 50].

To prepare the HG8 cell line deficient in HIF-1α, HG8 cells grown to confluency in ES medium in a 60mm cell culture dish were transfected with 2ug of HIF-1α Double Nickase Plasmid (sc-420856-NIC, Santa Cruz Biotechnology, USA), by means of Lipofectamine 2000 (Invitrogen, USA). After 5 h, the medium was changed for fresh ES medium supplemented by 5 μg/ml puromycine. After 24 h of selection, the medium was exchanged again for fresh ES medium. After 4 days, single clones were picked, trypsinized, and propagated. The successful generation of HG8 HIF-1α^−/−^ lines was confirmed by western blot analysis of cells under conditions stabilizing HIF-1α.

For the induction of differentiation into cardiac-like cells, cells were transferred onto agar-coated cell culture dishes for 5 days in ES medium without the addition of LIF to form embryoid bodies (EBs) [49]. On day 5, EBs were suspended in ITS medium (DMEM/F12, Insulin, Trasferine, Selenium (ITS), Invitrogen, USA; Penicilin/Streptomycin) and transferred onto a gelatin-coated culture surface inducing adhesion. R1 and R1^HIF-1α−/−^ cells were exposed to hypoxia on day 14 of differentiation for 24 hours. Purified cardiomyocytes (the selection cassette coding neomycin phosphotransferase enabling G418 resistance was driven by *Myh6* promoter) were prepared from differentiated HG8 cells as described earlier [49, 51]. Briefly, the cells were treated with G418 (0.5 mg/ml) on days 14 and 17. On day 20, cells were treated with 0.15 % collagenase and allowed to sediment several times to remove dead cell debris. The resulting pellet was then suspended in ES:ITS medium (1:1) seeded onto a gelatinized cell culture surface for 48 hours. After 48 hours, the medium was exchanged and the cells were treated with DMOG (0.5 mM) or cultivated in 1% O_2_ for 24 hours. In the case of rapamycin (Selleckchem, USA) pretreatment, the medium was exchanged for medium containing rapamycin (10 ng/ml) for the first 8 hours, and then exchanged again for fresh medium without rapamycin. Samples were collected after a total of 24 hours of treatment (the time-line of the cultivation is depicted in Figure 1A (HG8) and 1B (R1)).

### Animals and explanted mouse fetal hearts

C57BL/6 mice were obtained from the Laboratory Animal Breeding and Experimental Facility of the Faculty of Medicine, Masaryk University, Brno, Czech Republic. The mice were kept under controlled conditions; a standardized pelleted diet and HCl or UV light-treated tap water were available *ad libitum*. Experiments were performed in accordance with national and international guidelines on laboratory animal care and with the approval of the Institute's Ethical Committee conforming to the guidelines from Directive 2010/63/EU of the European Parliament on the protection of animals used for scientific purposes.

Pregnant mice were euthanized 13.5 days *post coitum* (dpc) by gradually filling the chamber with CO_2_. Unless otherwise stated, mouse fetal hearts were extracted from mouse embryos, washed in PBS, and either immediately frozen (0 h samples) or incubated on 0.1% bovine skin gelatin-coated cell culture plastic in high glucose Dulbecco's modified Eagle's medium (PAA Laboratories, Austria) supplemented with 100 IU/ml of penicillin, 0.1 mg/ml of streptomycin (Sigma-Aldrich, USA), and 10% FBS (PAA Laboratories, Austria) under various conditions and for various time periods (Figure 1A). For particular experiments DMOG (Calbiochem, USA) at a final concentration of 0.5 mM was added to cultivation media.

### RNA isolation and qRT-PCR

ES cells were washed in PBS and lysed in lysis buffer (UltraClean^®^ Tissue & Cells RNA Isolation Kit; MO BIO Laboratories, USA). Hearts were snap-frozen in liquid nitrogen either immediately after extraction or after *in vitro* incubation. The hearts were then disrupted in 200 μl lysis buffer (High Pure RNA Kit, Roche, Germany). Total RNA was isolated according to the kit manufacturer's recommendations. The concentration and purity of isolated RNA was assessed using a Nanodrop 1000 instrument (Thermo Scientific, USA) and only samples with an A280/A260 absorbance ratio greater than 1.9 were used for further investigation. 45 ng of total RNA was reverse transcribed in a 20 μl reaction mixture using Sensiscript Reverse Transcription kit (Qiagen, Germany), Anchored-Oligo (dt)18 Primer (1 μM final concentration), Random Hexamer Primer (10 μM final concentration), and Protector RNase Inhibitor (10 units/20 μl reaction) (all from Roche, Germany). Relative mRNA levels of genes encoding MHCα (*Myh6*), MHCβ (*Myh7*), Vascular endothelial growth factor (*Vegf*), Glucose transporter 1 (*Glut1*), Carbonic anhydrase (*CaIX*), sarcomeric α actinin (*Actn2*), and lncRNA *Mhrt* were analyzed, with Actn2 serving as reference genes. A reaction using the Universal Probe Library System and a LightCycler Probe Master was performed in a total volume of 10 μl on a LightCycler 480 Instrument (Roche, Germany). Specific combinations of primers and probes were designed using the Assay Design Center on the manufacturer's web pages; primers and probes were used at final concentrations of 10 μM and 0.5 μM, respectively. For the specific combinations of primers and UPL probes, see Table 1. The cycling program was set as follows: 95°C for 10 min followed by 45 cycles of 95°C for 10 s, 60°C for 30 s, and 72°C for 1 s, with data acquisition. Results are expressed as the fold difference between the target gene and the reference gene, determined by the relative quantification 2^−ΔCq^ method. Data are presented as 2^-(Cq(target)-Cq(reference))^ ± SEM. The mRNA expression profiles of *Myh6* and *Myh7* genes were also verified using TaqManGene Expression Assay (Applied Biosytems, USA).

**Table 1:** Specific combinations of primers and UPL probes

Target	Left primer 5′→3′	Right primer 5′→3′	UPL probe
*Actn2*	CCGGATTCTGGCTTCTGAT	GAGGCAGCTCTCGACGAA	53
*Myh6*	CGCATCAAGGAGCTCACC	CCTGCAGCCGCATTAAGT	6
*Myh7*	CGCATCAAGGAGCTCACC	CTGCAGCCGCAGTAGGTT	6
*Vegf*	CAGGCTGCTGTAACGATGAA	GCTTTGGTGAGGTTTGATCC	9
*Glut 1*	GGACCCTGCACCTCATTG	GCCACGATGCTCAGATAGG	20
*CaIX*	ATTCCTGCTTCACTGCTGGT	CTTTGGTCCCACTTCTGTGC	16
*Mhrt*	ACACAGATGGACGCTCTGG	GGATGAGGCGGAGGAGAG	95

### Antisense *Myh7* transcript analysis

The expression of mouse antisense *Myh7* transcript was analyzed using forward 5′-AGCCAGGCCTGCTACCAAAGACACC-3′ and reverse 5′-CCCTAGAACACCACAGAGCATAAA-3′ primers designed according to Haddad [28]. Briefly, total RNA was isolated as described above and equal amounts of total RNA were reverse-transcribed by means of Sensiscript Reverse Transcription kit (Qiagen, Germany) using only forward primer in the reaction mixture. 3 μl of 4 × diluted resulting specific cDNA was subsequently amplified in 30 cycles of PCR using Hot Start Titanium Taq Polymerase kit (Clontech, USA) employing forward and reverse primers [28]. The PCR conditions were set as follows according to the polymerase kit manufacturer's recommendations: 95°C for 5 min followed by 30 cycles of 95°C for 30 s and 68°C for 20 s, ending the reaction with 68°C for 1 min. The PCR product was visualized on 2% agarose gel supplemented with ethidium bromide. As a negative control, a sample resulting from reverse-transcription with no forward primer was used as a template in the amplification step [40].

### Western blot analysis

Cell sample harvesting and immunoblot analysis were performed as presented previously [50]. To detect proteins, the primary antibodies β-actin (sc-47778, Santa Cruz Biotechnology, USA), vinculin (Sigma-Aldrich), HIF-1α (GTX127309, Genetex, USA), HIF-2α (NB100–122, Novus Biologicals, USA or GTX30114, Genetex, USA), p70 s6 kinase and phospho-p70 s6 kinase (Thr 389) (9202 and 9205 respectively, both from Cell Signaling, USA), and MF20 (a hybridoma producing both antibody detecting α and β MHC isoforms; Developmental Studies Hybridoma Bank, Iowa City, IA, USA) were used in 5% non-fat milk/TBS-T at 4°C overnight. Corresponding secondary HRP-conjugated anti-rabbit or anti-mouse antibodies (in 5% non-fat milk/TBS-T, 1 h, RT; Cell Signaling, USA) were employed. Immunoreactive bands were detected using ECL detection reagent kit (Pierce, USA) and exposed to radiographic film (AGFA, Belgium). The adjustment of brightness and contrast was applied to the whole image.

### *In silico* analysis of the DNA locus harbouring *Myh6/7* genes

The coding DNA sequences of mouse *Myh6* and *Myh7* genes, the intergenic regions, and the flanking regions located 30 kb upstream and downstream of both 5′ and 3′ ends were analyzed for the presence of potential HIF binding sites (HREs); for further analysis, only 10 kb flanking regions were considered. The genomic sequence was sourced by the Ensembl genome browser and screened for HIF-1, HIF-1 ancillary sequence, and HIF1beta/ARNT by means of the following online tools using different algorithms for the prediction of the presence of transcription factor binding sites: MatInspector (genomatix.de) [52], Match, Patch, P-Match [53, 54], and AliBaba2 (gene-regulation.com) [55]. For the analysis of evolutionarily conserved putative HREs, the rVista 2.0 tool (rvista.dcode.org) was employed as described previously [56]. Predicted HREs containing the [A/G]CGT core motif were further screened and scored by the position-specific matrix of frequencies according to recommendations described earlier [57]. The calculated score (Table 2.) reflects the similarity of the analyzed sequence to known functional hypoxia-responsive sequences.

**Table 2:** Primers used for the amplification of immunoprecipitated DNA and the similarity of individual predicted binding sites to functional HRE (Score)

Target	Left primer 5′→3′	Right primer 5′→3′	Score
*Cluster 9*	AAAGCATCCCAGGAAGAAAGCAGCA	AGTCGAGACCCACAGGCTGAGAACC	7.15
*Cluster 11*	AATCTGACACTACCGGCCACCTGCT	ACCCCAGCACTCCTGTGGCAAAATA	7.72/7.58
*Cluster 13a*	CGTAGGAATGTGGGAGCCCAAGTGT	AGCCTCATGATTCCAACAGCCCTCT	6.34/7.84
*Cluster 13b*	TCAGAGGGCTGTTGGAATCATGAGG	AGAAAGGAACCTGCCAGGCTTTGG	8.85
*Cluster 19*	TATCCACAGTGTCCCTTGCCCCTTC	TGGTCAGAACACAGAGGGCTGTTGA	8.33
*Intron 25–26*	ACTCCCGTGCCATTCTGCCCTAGTAA	GACCAGCCATGAACCACAAGGTAGGA	7.34
*Glut1*	TCAGCAAAGCCTTTTCAAGCCCTACC	CCCTGGAGGTGACTTCAGCCCTTAAA	7.79

### Chromatin immunoprecipitation (ChIP)

ChIP assay was performed on 10-day differentiated R1 exposed to 1% O_2_ for 24 h using 2 ug of HIF-1α specific antibody (GTX127309 Genetex, USA) according to a previously described protocol [58]. An alternative protocol using ExactaChIP Human/Mouse HIF-1α Chromatin Immunoprecipitation Kit (R&D Systems USA) was applied according to the manufacturer's recommendations in embryonal hearts exposed to 1% O_2_ for 24 h (data not shown). Immunoprecipitated DNA was purified using QIAquick PCR Purification Kit (Qiagen, Germany) and subsequently amplified in a PCR step using Titanium TaqPCR Kit (Clontech Laboratories, Inc., USA) with primer sets corresponding to putative HIF-binding DNA clusters predicted in the *Myh7*/*Myh6* encoding locus (Table 2). The products were separated and visualized by means of EtBr-based agarose electrophoresis. As a positive control, the presence of Glut1 enhancer sequence 5′-CACAGGCGTGCCGTCTGACACGCA-3′ [59] in the HIF-1α-immunoprecipitated DNA sample was analyzed (primers listed in Table 2).

### Statistical analysis

Unless otherwise stated, data are presented as the average of at least 3 independent measurements ± SEM. Groups were compared using one-way ANOVA with the Tukey (HSD) post hoc test or with the Fisher's LSD post hoc test. Values of *p* equal to, or less than 0.05 were considered significant and are marked with asterisks or a cross symbol.

## SUPPLEMENTARY MATERIALS FIGURES



## Abbreviations

DMEM: Dulbecco's modified Eagle's medium
DMOG: dimethyloxalylglycine
HIF: hypoxia inducible factor
HRE: HIF response element
ChIP: Chromatin immunoprecipitation
lncRNA: long noncoding RNA
mESC: mouse embryonic stem cell
MHC: myosin heavy chain
mTOR: mammalian (mechanistic) target of rapamycin
mTORC1: mTOR complex 1
PHD: prolyl hydroxylase domain enzyme
