# Supplementary material for: Hypoxia favors myosin heavy chain beta gene expression in an Hif-1alpha-dependent manner
Source: Oncotarget. 2017 Jul 5;8(48):83684–97. doi: 10.18632/oncotarget.19016 (PMC5663546; doi:10.18632/oncotarget.19016)
Supplement: Supplementary file 1 [file oncotarget-08-83684-s001.pdf]

Hypoxia favors myosin heavy chain beta gene expression in an Hif-1alpha-dependent manner

Supplementary Materials

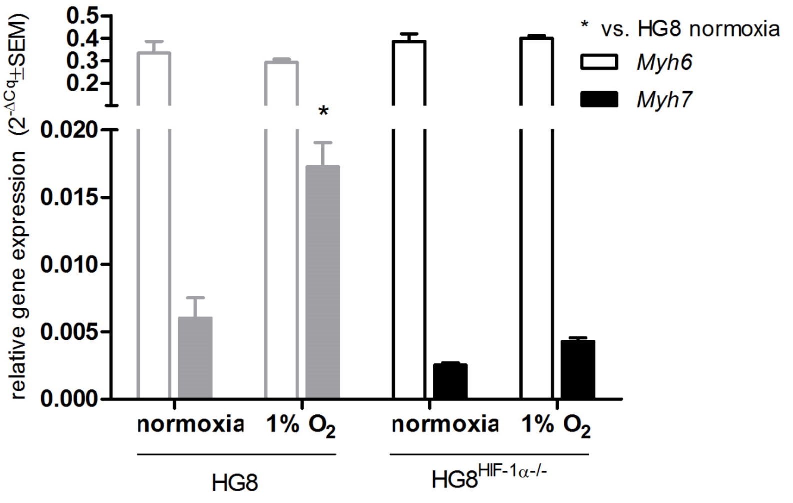

Supplementary Figure 1: Effect of hypoxia and the pharmacological stabilization of HIF on MHCs expression. *Myh6* and *Myh7* relative mRNA levels in cardiomyocytes derived from parental HG8 and the second clone of HG8$^{HIF\text{-}1\alpha-/-}$ mESC (n = 3); same data depicted in Fig. 1B are displayed in grey for comparison. Data are presented as $2^{-\Delta Cq}$ ± SEM (groups were compared using one-way ANOVA with the Tukey (HSD) post hoc test $*p < 0.05$).

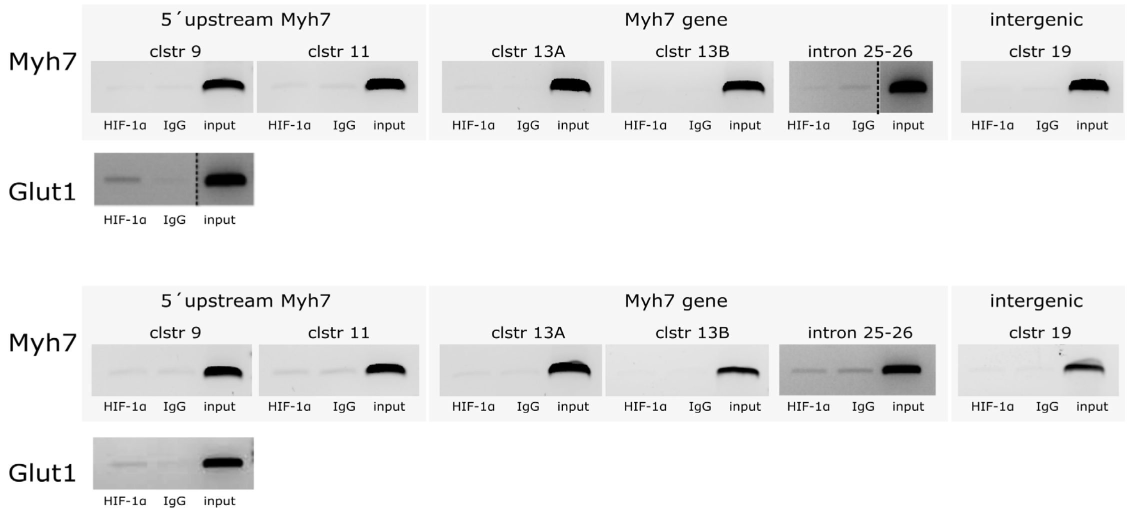

Supplementary Figure 2: Analysis of HIF-1α binding to the DNA locus encoding MHCα and MHCβ. Additional repetitions of ChIP analysis of HIF-1α binding to predicted binding sites performed on samples from 10 day-differentiated R1 mESCs exposed to 1% O_2 hypoxia for 24 h. HIF-1α binding to its target gene Glut1 served as ChIP assay positive control (cropped representative gels are shown).

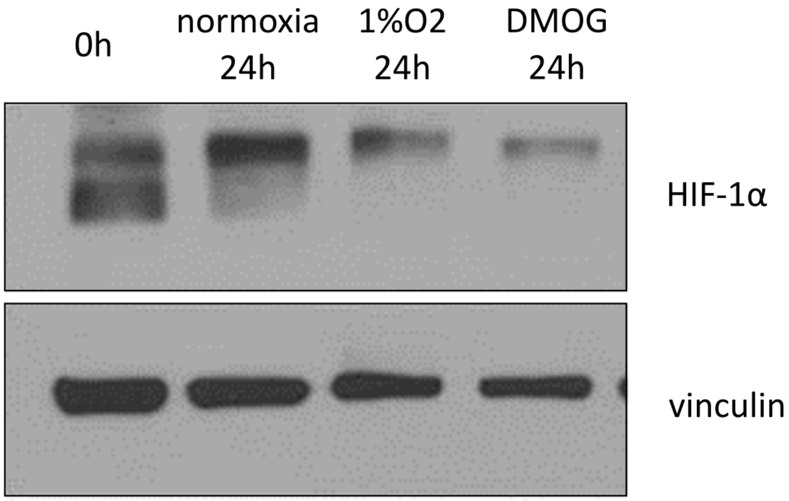

Supplementary Figure 3: Levels of HIF-1α protein in mouse fetal heart model after 24 h incubation in hypoxia and the pharmacological stabilization of HIF. Stabilization of HIF-1α protein after 24 h of *in vitro* culture in normoxia, 1% O_2 and DMOG presence (cropped representative western blots are shown, *n* = 4).
